# Joint Optimization of User Association and Dynamic Multi-UAV Deployment for Maritime Emergency Communications

**DOI:** 10.3390/e28050561

**Published:** 2026-05-17

**Authors:** Xiaonan Ma, Hua Yang, Yanli Xu, Naoki Wakamiya

**Affiliations:** 1 College of Information Engineering, Shanghai Maritime University, Shanghai 201306, China; 15562752568@163.com (X.M.); ylxu@shmtu.edu.cn (Y.X.); 2 Graduate School of Information Science and Technology, The University of Osaka, 1-5 Yamadaoka, Suita 565-0871, Osaka, Japan; wakamiya@ist.osaka-u.ac.jp

**Keywords:** maritime emergency communications, UAV base station, priority-aware user association, dynamic deployment, branch-and-cut, multi-agent reinforcement learning

## Abstract

Maritime emergency response requires broadband and reliable communications in sea areas where shore coverage is limited or emergency connectivity is temporarily unavailable, making rapid on-demand aerial networking essential. Unmanned aerial vehicles (UAVs) acting as aerial base stations can be rapidly deployed to provide on-demand coverage; however, ship mobility, heterogeneous emergency priorities, and UAV endurance limitations make the joint optimization of user association and multi-UAV deployment a challenging mixed-integer, long-horizon decision problem. This paper considers a multi-UAV maritime emergency communication system where ships are categorized into multiple priority classes and served links must satisfy a minimum signal-to-noise ratio (SNR) constraint. We formulate a long-term system-utility maximization problem that jointly determines (i) per-slot association between UAVs and ships under capacity, priority, and SNR constraints, and (ii) dynamic UAV deployment under mobility, geofencing, and battery constraints. To obtain tractable and high-quality solutions, we decompose the problem into two coupled subproblems. For user association, we propose a Priority-Aware Branch-and-Cut (PA-BAC) algorithm that integrates linear programming relaxation, cutting-plane tightening, and priority-guided branching, with a priority-greedy feasible initialization to accelerate incumbent improvement. For dynamic deployment, we develop an Enhanced Multi-Agent Proximal Policy Optimization (E-MAPPO) method featuring a global value network, entropy regularization, and sequential actor updates to enhance learning stability and exploration. Importantly, the PA-BAC association is embedded into the learning loop to provide reliable, constraint-satisfying per-slot rewards and reduce the burden of end-to-end learning over hybrid-action spaces. Simulation results demonstrate that PA-BAC consistently improves normalized priority-weighted throughput over heuristic association baselines. Moreover, by mathematically enforcing priority and QoS feasibility at every slot and delegating only continuous mobility to MARL, the integrated E-MAPPO-PA-BAC framework achieves higher long-term system utility, improved energy efficiency, and strong robustness across varying ship densities—properties that are vital for time-sensitive maritime emergency communications. Additional runtime, sensitivity, and AIS-driven trace evaluations further verify the computational practicality of PA-BAC and the applicability of the proposed framework under realistic ship mobility patterns.

## 1. Introduction

When coastal disasters, ship collisions, or extreme weather incidents occur at sea, communication services in the affected maritime areas often become unavailable due to limited shore coverage, disrupted backhaul connectivity, or overloaded vessel-mounted systems. In this paper, we focus on a UAV-enabled maritime emergency network where rotary-wing UAVs serve as the primary aerial base stations to provide rapid on-demand coverage for ships, without relying on terrestrial base stations. Recent surveys on maritime communications and UAV-aided maritime networking emphasize that large ocean areas still suffer from intermittent coverage and that aerial platforms are increasingly viewed as agile complements to satellite and terrestrial systems, especially for coverage gaps and emergency response operations [1,2,3]. Meanwhile, broader UAV-assisted wireless communication surveys also highlight the maturity of UAV-enabled networking concepts such as aerial base stations, relaying, and on-demand coverage, while underscoring that practical deployment in dynamic and heterogeneous environments remains challenging [4,5,6].

Maritime emergency networks differ from conventional terrestrial UAV-aided cellular scenarios in several fundamental ways. First, user mobility is persistent and mission-driven: vessels continue moving according to navigation routes, rescue patterns, or stochastic drift under wind and currents; thus, coverage demands evolve continuously across time rather than remaining quasi-static [1,2,3]. Second, the service urgency varies across users. In a maritime emergency, distress vessels often require strict minimum service guarantees, while command and rescue vessels and supporting or task-oriented ships demand differentiated QoS levels. This heterogeneity motivates priority-aware designs rather than one-size-fits-all throughput maximization [6]. Third, the aerial segment is energy-constrained. UAV base stations (UAV-BSs) must balance communication performance with propulsion energy and mobility costs; energy efficiency and long-horizon endurance are therefore essential considerations in emergency deployment [4,7]. Finally, maritime connectivity is influenced by long-range links, sparse user distributions, and the need for rapid reconfiguration over open-sea areas, which complicates both modeling and online control [1,2,3]. Additionally, real-world deployment in maritime emergency scenarios presents extra challenges due to limited infrastructure support and more difficult coordination. These factors mean that not only algorithm performance must be considered, but also the feasibility of implementation in dynamic maritime environments.

A natural approach is to jointly optimize UAV deployment and trajectory control over time along with user association, which determines the serving UAV for each ship at each slot. However, this joint design is intrinsically difficult because it combines continuous decisions regarding UAV positions and velocities with discrete binary decisions representing association indicators. Furthermore, it must satisfy hard per-slot constraints such as UAV capacity and minimum-rate requirements for high-priority users. This leads to a large-scale, mixed-integer, time-coupled optimization problem that is computationally challenging for real-time maritime emergencies. On the one hand, mathematical optimization models can explicitly encode constraints and yield feasible or optimal association decisions when UAV positions are fixed. For example, on-demand UAV-BS deployment has been formulated to provide full coverage under demand dynamics, demonstrating the potential of optimization-style modeling for feasibility and service assurance [8]. On the other hand, pure optimization becomes increasingly costly when extended to multi-slot trajectory control with mobility coupling and UAV battery constraints, especially when the environment is uncertain and vessel dynamics are time-varying.

To address uncertainty and long-horizon coupling, learning-based control—particularly deep reinforcement learning (DRL) and multi-agent reinforcement learning (MARL)—has become an active research direction for UAV deployment and emergency networking. Recent works show that DRL can be used to maximize coverage or utility in emergency UAV communication settings, offering adaptive policies that respond to mobility and demand changes [9,10]. In addition, energy-oriented DRL designs have been explored to improve energy efficiency in UAV-assisted networks, which is especially relevant in maritime operations where endurance is critical [7]. These trends suggest that learning-based approaches are promising for dynamic maritime emergencies.

Nevertheless, two gaps remain particularly important for priority-sensitive maritime emergency communications. First, many end-to-end DRL and MARL approaches treat association decisions as part of the policy output. While this can improve scalability, it can also lead to infeasible or suboptimal assignments when strict per-slot capacity and QoS constraints must be satisfied—especially when high-priority users require guaranteed service. Second, purely optimization-based association, even if optimal for a single slot, does not directly resolve the long-horizon coupling induced by UAV mobility and energy budgets since decisions made in the moment affect future reachable positions and remaining battery. In recent DRL formulations that jointly optimize trajectory control, resource allocation, and user association for multi-UAV networks, the hybrid discrete–continuous nature of decisions remains evident and can complicate stable learning and constraint satisfaction [11]. In maritime emergency networks, these issues are amplified by persistent ship mobility and heterogeneous priority demands.

Motivated by these observations, we pursue a hybrid optimization-and-learning framework for maritime emergency communications. Considering that the association strategies at different time steps are independent despite the correlation between deployment and association variables, we decompose the optimization problem into two subproblems. The key idea is to first determine the optimal association strategy based on the optimal UAV deployment positions per slot using our proposed Priority-Aware Branch-and-Cut (PA-BAC) algorithm, so that priority and QoS constraints are enforced by construction. Then, under the optimal association strategy, we find the optimal UAV deployment positions over time via the Enhanced Multi-Agent Proximal Policy Optimization with PA-BAC (E-MAPPO-PA-BAC) algorithm. This algorithm is based on centralized training with decentralized execution and on-policy rollouts, where the PA-BAC module is invoked at each rollout step. By embedding a constraint-satisfying per-slot association solver inside MARL rollouts, we decouple discrete assignment complexity from continuous mobility control, improving feasibility and interpretability in priority-sensitive settings. In particular, our objective explicitly maximizes the long-term system utility, defined by the coverage rate and the normalized priority-weighted throughput, while managing UAV energy consumption and boundary limits through physical constraints or reinforcement learning penalty terms. In summary, our contributions are:

1. We formulate a priority-aware joint user association and dynamic multi-UAV deployment problem for maritime emergency communications by jointly considering heterogeneous ship priorities, ship mobility, UAV capacity, SNR feasibility, mobility boundaries, and energy consumption within a unified long-horizon optimization framework. Unlike conventional UAV-enabled emergency communication studies that mainly focus on coverage enhancement or deployment optimization alone, the proposed formulation explicitly captures the strong coupling between discrete UAV–ship association decisions and continuous UAV mobility control in time-varying maritime scenarios. By defining the system utility as a combination of coverage ratio and normalized priority-weighted throughput, the formulation reflects both broad connectivity restoration and differentiated service protection for high-priority emergency users. This leads to a challenging mixed-integer, long-horizon decision problem with coupled discrete–continuous variables, for which a direct end-to-end solution is computationally difficult in practical maritime emergency operations.
2. We develop a Priority-Aware Branch-and-Cut algorithm, termed PA-BAC, to solve the per-slot user association subproblem under strict capacity, priority-service, and SNR-feasibility constraints. Rather than relying on myopic association heuristics or learning-based association outputs that may violate hard service constraints, PA-BAC formulates the association decision as a binary integer linear programming problem and solves it through LP relaxation, cutting-plane tightening, and priority-guided branching. In particular, SNR-based link screening removes infeasible UAV–ship pairs before optimization, while priority-greedy initialization and priority-guided branching embed maritime emergency priorities into the search process. As a result, PA-BAC can provide constraint-satisfying and priority-consistent association decisions at each time slot, thereby protecting high-urgency ships while efficiently utilizing limited UAV service capacity.
3. We propose a hybrid optimization-and-learning framework, termed E-MAPPO-PA-BAC, for dynamic multi-UAV deployment in maritime emergency communication networks. Instead of treating user association and UAV movement as a single hybrid-action space to be learned end-to-end, the proposed framework decomposes the original problem into two coordinated decision layers: PA-BAC handles the discrete, priority-aware, constraint-satisfying association decision, while E-MAPPO learns continuous UAV mobility policies. Within the continuous-control learning layer, the centralized critic, entropy regularization, and sequential actor updates are used as stabilization mechanisms for cooperative multi-UAV policy learning. By embedding PA-BAC into the MARL rollout process, the deployment policy receives rewards evaluated under feasible and priority-aware association decisions, which reduces the learning burden caused by hybrid-action spaces and improves the interpretability and reliability of the resulting multi-UAV control policy.

## 2. Related Work

This section reviews prior work in categories: UAV-enabled emergency communications, optimization-based UAV deployment and placement, learning-based joint deployment and association, maritime-oriented UAV networking and sensing–communication co-design, and MARL and hybrid-action learning foundations. We then position our work relative to these studies and highlight the gap addressed by our hybrid design.

### 2.1. UAV-Enabled Emergency Communications

A large body of work investigates UAVs as rapidly deployable communication infrastructure for disaster relief and emergency response. Bilgehan and Sabuncu proposed an adaptive UAV deployment method for disaster-stricken emergency networks using a multi-objective optimization approach to enhance connectivity; however, the formulation is not tailored to maritime mobility patterns and does not explicitly emphasize strict priority–guarantee association under per-slot constraints [9]. Zhao, Liu, and Shang investigated coverage maximization in UAV-based emergency communication networks using deep reinforcement learning, demonstrating that DRL can adapt UAV actions to improve coverage performance; yet, association feasibility under strict priority and QoS constraints remains difficult when association is implicitly learned or when decision spaces are hybrid [10]. Sun et al. studied energy-efficiency maximization for WPT-enabled UAV-assisted emergency communication with user mobility, highlighting that mobility and energy objectives must be jointly considered; nonetheless, the work is not specialized for priority-stratified maritime rescue fleets and does not focus on hard constraint enforcement via optimal association [12].

From a resource management perspective, Wang et al. presented DRL-based resource scheduling scheme for UAV-assisted emergency communication networks, illustrating the potential of learning-based scheduling in emergency scenarios. However, resource scheduling alone does not resolve the discrete association feasibility issue under strict per-slot guarantees, especially when multiple UAVs must coordinate over time [13]. In addition, Zhang et al. investigated joint task scheduling and multi-UAV deployment for aerial computing in emergency communication networks, showing that emergency systems often involve coupled computing and communication objectives; still, translating such ideas to maritime emergency communication with strict priority-aware association and long-horizon UAV energy coupling remains nontrivial [14]. Overall, these studies confirm the value of adaptive UAV control for emergency communications, but leave largely unexplored the reliable handling of priority-aware service guarantees and hybrid discrete–continuous decisions in maritime time-varying environments.

### 2.2. Optimization-Based UAV Placement and Deployment

Another major research direction formulates UAV placement, deployment, and association as mathematical optimization problems. Premkumar and Van Scoy formulated optimal UAV base station positioning using mixed-integer linear programming (MILP), providing a clear optimization framework for UAV-BS placement; however, MILP formulations can become computationally expensive when extended to multi-slot dynamic trajectories and large-scale online maritime rescue operations [15]. Shojaei et al. proposed a fairness-aware placement framework for multiple aerial base stations, demonstrating that fairness objectives can significantly alter placement decisions; nevertheless, fairness objectives do not automatically guarantee strict priority service under hard per-slot constraints, and the work does not focus on maritime mobility coupling [16]. Zhu et al. developed multi-objective deployment optimization for UAVs toward energy-efficient wireless coverage, emphasizing the tradeoff between coverage and energy; still, extending multi-objective optimization to time-coupled multi-agent trajectories with strict association feasibility can be challenging in rapidly evolving scenarios [17].

Heuristic and metaheuristic approaches have also been adopted for joint placement and association. Chin et al. investigated joint aerial base station placement and user association using a whale optimization approach, indicating that population-based heuristics can handle larger instances than exact solvers in some settings; however, heuristic methods generally lack hard feasibility guarantees for priority users and may be sensitive to hyperparameters or scenario shifts [18]. Classical placement works remain foundational: Lyu et al. studied placement optimization of UAV-mounted mobile base stations, offering early insights into UAV-BS positioning; yet, these results do not directly address multi-agent long-horizon maritime emergency coupling and strict per-slot association constraints [19].

In our context, optimization plays a different role: instead of attempting to solve the entire long-horizon mixed-integer trajectory and association problem online, we use optimization selectively to solve the per-slot association problem optimally given the optimal UAV deployment positions. Specifically, we achieve this by constructing a BILP and solving it with the proposed PA-BAC algorithm. This perspective allows us to enforce priority and QoS constraints through construction while delegating time-coupled mobility control to learning.

### 2.3. Learning-Based Joint Deployment and Association

A growing body of works uses DRL and MARL to jointly optimize UAV deployment and association in dynamic environments. Wang et al. proposed CNN-based rapid deployment for emergency integrated sensing and communication networks, directly addressing the complexity of emergency network configuration [20]. Guo et al. studied time-efficient UAV deployment using an improved virtual force approach, reflecting the trend toward heuristic methods for reducing computational complexity [21]. These works motivate our direction but also highlight the challenge of ensuring hard constraints under hybrid action spaces.

In cooperative multi-UAV control, MARL methods have been applied to related problems such as fairness-aware communications or coverage. Luo et al. proposed a multi-agent deep reinforcement learning method for UAV-assisted fair communications for multi-pair users, showing that MARL can coordinate UAV behaviors to improve fairness-related objectives [22]. Xiao et al. presented a distributed multi-UAV dynamic area coverage algorithm based on deep reinforcement learning for complex environments, showing the ability of DRL to coordinate multi-UAV coverage; yet, area coverage does not directly incorporate discrete association feasibility with QoS constraints and priority requirements typical of maritime emergency communications [23].

For association and resource allocation in UAV-assisted cellular settings, Song et al. proposed a multi-agent PPO-based approach for efficient user association and resource allocation in UAV-assisted heterogeneous cellular networks, illustrating that policy-gradient MARL can scale to joint association and resource decisions; however, training a policy to output discrete association decisions still risks constraint violations unless combined with explicit feasibility mechanisms, especially under strict priority guarantees [24]. In contrast, our framework removes this risk by optimizing association per slot to ensure feasibility by construction while using MARL for continuous deployment and trajectory control.

### 2.4. Maritime-Oriented UAV Networking and Sensing–Communication Co-Design

Beyond terrestrial settings, surveys on maritime communications emphasize that maritime connectivity involves unique challenges: long-range coverage gaps, sparse users, and fast-changing requirements in emergencies [1,2,3]. Recent research has also increasingly explored sensing–communication co-design in UAV cellular networks, which is relevant for emergency response, such as simultaneous sensing of disaster areas and communication provisioning. Diaz-Vilor et al. studied sensing and communication in UAV cellular networks with a focus on design and optimization, reflecting the broader move toward integrated multi-function UAV networks; yet, maritime emergency networks with strict priority-aware association remain underexplored in such co-design studies [25]. For rapid emergency deployment, Wang et al. proposed a CNN-based rapid deployment approach for UAV-assisted emergency integrated sensing and communication networks, emphasizing fast configuration; however, rapid deployment alone does not solve the long-horizon multi-UAV movement problem with discrete association feasibility for high-priority users [20]. These directions are complementary. Our work focuses on priority-aware communication service continuity through hybrid optimization and learning, and can be combined with sensing and computation modules in future extensions.

### 2.5. MARL and Hybrid-Action Learning Foundations

Our problem features a hybrid decision structure: association is discrete, while UAV mobility control is continuous. Reinforcement learning with hybrid-action spaces has been proposed to address such discrete–continuous control. Xu et al. proposed an action-decoupled SAC approach for discrete–continuous hybrid-action spaces, explicitly targeting the learning difficulty induced by mixed action types; nevertheless, even with decoupled hybrid-action learning, guaranteeing strict feasibility of association under hard QoS constraints remains challenging without an explicit optimization layer [26]. Si et al. studied UAV-assisted semantic communication with hybrid-action reinforcement learning, further confirming that hybrid-action formulations naturally arise in UAV communication problems; however, the focus differs from our maritime emergency objective with strict priority–guarantee association solved optimally per slot [27].

From a broader perspective, surveys on MARL summarize algorithmic families, stability issues, and the growing use of MARL in engineering applications. Ning and Xie provided a survey on multi-agent reinforcement learning and its application, highlighting the breadth of MARL methods and the importance of careful problem modeling [28]. In policy-gradient methods, PPO is a widely used baseline due to its clipped objective and empirical robustness [29]. Moreover, empirical studies have examined PPO’s performance in cooperative multi-agent games, reinforcing the appeal of PPO-style training for cooperative settings [30]. These works support our choice of a PPO-style cooperative MARL backbone for learning multi-UAV deployment policies while keeping the discrete association decisions outside the policy to preserve feasibility.

In summary, existing studies have established three important foundations for UAV-enabled emergency communications: optimization-based methods can explicitly model placement and association constraints; DRL/MARL methods can adapt UAV deployment to dynamic environments; and hybrid-action learning provides useful tools for mixed discrete–continuous decision problems. However, these directions are usually developed separately. End-to-end learning methods can adapt to dynamic deployment but do not explicitly guarantee priority-aware association feasibility, whereas exact optimization methods can enforce constraints but are difficult to apply directly to long-horizon multi-UAV trajectory control. Different from these studies, our framework exploits the problem structure by assigning the binary association decision to a priority-aware optimization layer and the continuous UAV mobility decision to MARL. The resulting E-MAPPO-PA-BAC framework explicitly integrates constraint-satisfying per-slot association with adaptive long-horizon multi-UAV deployment. This integration constitutes the main novelty of this work.

## 3. System Model

### 3.1. Network Model

**Figure 1:**
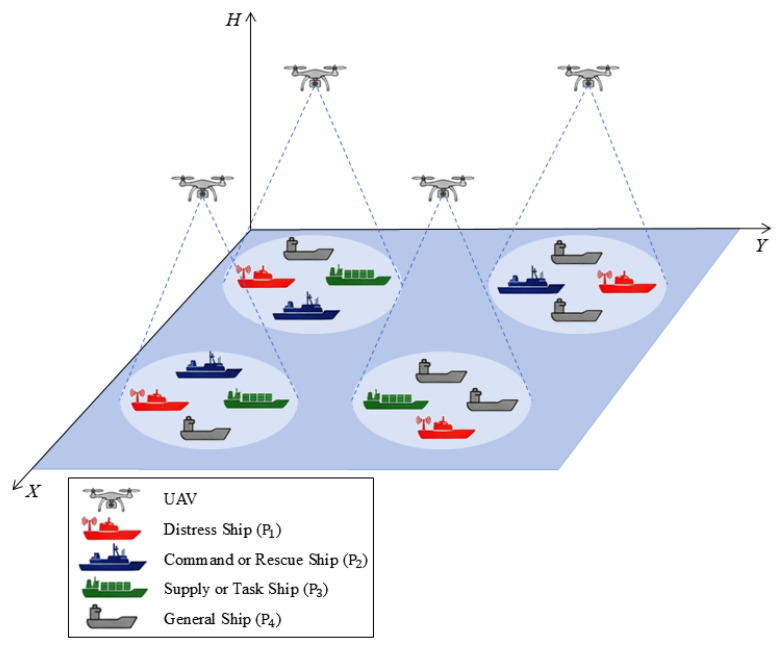
Maritime emergency communication network model.

We consider a time-critical maritime emergency communication scenario within a sea area denoted by *Z*. To restore connectivity, a fleet of rotary-wing UAVs are deployed as aerial base stations (UAV-BSs). We adopt a discrete-time model. The mission horizon is divided into equal-length time slots of duration Δt. Let T={1,2,…,T}, and we set Δt=1 s. We further assume slot-level time synchronization among UAVs and ships. In maritime emergency operations, such synchronization can be supported by a common GNSS time reference, satellite-assisted timing, or periodic timing beacons broadcast by a command vessel. Since the slot duration is set to Δt=1 s, the residual synchronization error is much smaller than the decision interval and is therefore neglected in this work. As shown in Figure 1, the system includes UAV base stations and maritime users. Let *M* be the number of UAVs, and define the UAV set $M=\{b_{1},b_{2},\ldots ,b_{M}\}$. For UAV $b_{m}$, its 3D position at slot *t* is $p_{b_{m}}\left(t\right)=\left(x_{b_{m}}\left(t\right),y_{b_{m}}\left(t\right),h\right)$, ∀m=1,2,…,M. Here, $\left(x_{b_{m}}\left(t\right),y_{b_{m}}\left(t\right)\right)$ is the horizontal projection on the sea surface. All UAVs fly at a fixed altitude *h*, which is practical for safety, regulation compliance, and stable coverage. Let *N* be the number of ships, and define the user set $N=\{u_{1},u_{2},\ldots ,u_{N}\}$. The position of user $u_{n}$ at slot *t* is $p_{u_{n}}\left(t\right)=\left(x_{u_{n}}\left(t\right),y_{u_{n}}\left(t\right),0\right)$, ∀n=1,2,…,N. We define a binary association decision variable $\alpha_{m,n}\left(t\right)$ as

(1) $$\alpha_{m,n}\left(t\right)=\left\{\begin{array}{cc} 1, & \mathrm{if}\;\mathrm{ship}\;u_{n}\;\mathrm{is}\;\mathrm{associated}\;\mathrm{with}\;\mathrm{UAV}\;b_{m}\;\mathrm{at}\;\mathrm{slot}\;t,\\ 0, & \mathrm{otherwise}. \end{array}\right.$$

This variable will be optimized subject to connectivity and UAV capacity constraints.

### 3.2. User Priority Stratification

In maritime emergency operations, users may have different levels of service urgency. To reflect maritime rescue protocols, we divide N into four priority classes. Each user $u_{n}$ is assigned a priority weight $w(u_{n})$. Priority 1: Distress ships ($P_{1}$). These ships are directly in danger and require immediate protection. Let the set be $N_{P_{1}}$ with priority weight $w_{1}$. Priority 2: Command or rescue ships ($P_{2}$). These ships coordinate rescue missions and require high-reliability links. Let the set be $N_{P_{2}}$ with priority weight $w_{2}$. Priority 3: Supply or task ships ($P_{3}$). These ships provide supplies or perform supporting tasks. Let the set be $N_{P_{3}}$ with priority weight $w_{3}$. Priority 4: General ships ($P_{4}$). These ships have comparatively less urgent demands. Let the set be $N_{P_{4}}$ with priority weight $w_{4}$. The priority weights satisfy the strict ordering $w_{1}>w_{2}>w_{3}>w_{4}>0$. The subsets are mutually exclusive and collectively exhaustive: $N_{P_{1}}\cup N_{P_{2}}\cup N_{P_{3}}\cup N_{P_{4}}=N$, $N_{P_{i}}\cap N_{P_{j}}=⌀,\forall i\neq j$.

### 3.3. Communication Model

Maritime wireless channels are characterized by strong reflections and limited scattering. We adopt a probabilistic LoS model dependent on UAV–user geometry. The 3D distance between UAV $b_{m}$ and user $u_{n}$ at slot *t* is

(2) $$d_{b_{m},u_{n}}\left(t\right)=\sqrt{{\left(x_{b_{m}}\left(t\right)-x_{u_{n}}\left(t\right)\right)}^{2}+{\left(y_{b_{m}}\left(t\right)-y_{u_{n}}\left(t\right)\right)}^{2}+h^{2}}$$

The elevation angle is given by

(3) $$\theta_{b_{m},u_{n}}\left(t\right)=\arctan\left(\frac{h}{\sqrt{{\left(x_{b_{m}}\left(t\right)-x_{u_{n}}\left(t\right)\right)}^{2}+{\left(y_{b_{m}}\left(t\right)-y_{u_{n}}\left(t\right)\right)}^{2}}}\right)\cdot \frac{180}{\pi }$$

The LoS probability is modeled by a sigmoid function

(4) $$P_{b_{m},u_{n}}^{\mathrm{LoS}}\left(t\right)=\frac{1}{1+a\cdot \exp\left(-b(\theta_{b_{m},u_{n}}\left(t\right)-a)\right)}$$

where *a* and *b* are environment-dependent constants. Then

(5) $$P_{b_{m},u_{n}}^{\mathrm{NLoS}}\left(t\right)=1-P_{b_{m},u_{n}}^{\mathrm{LoS}}\left(t\right)$$

Path loss (dB) under LoS and NLoS is

(6) $$L_{b_{m},u_{n}}^{\mathrm{LoS}}\left(t\right)=20\log_{10}d_{b_{m},u_{n}}\left(t\right)+20\log_{10}f_{c}+20\log_{10}\left(\frac{4\pi }{c}\right)+\eta_{\mathrm{LoS}}$$

(7) $$L_{b_{m},u_{n}}^{\mathrm{NLoS}}\left(t\right)=20\log_{10}d_{b_{m},u_{n}}\left(t\right)+20\log_{10}f_{c}+20\log_{10}\left(\frac{4\pi }{c}\right)+\eta_{\mathrm{NLoS}}$$

where $f_{c}$ is carrier frequency, *c* is speed of light, and $\eta_{\mathrm{LoS}},\eta_{\mathrm{NLoS}}$ are additional loss factors. The expected path loss is

(8) $$L_{b_{m},u_{n}}\left(t\right)=P_{b_{m},u_{n}}^{\mathrm{LoS}}\left(t\right)L_{b_{m},u_{n}}^{\mathrm{LoS}}\left(t\right)+P_{b_{m},u_{n}}^{\mathrm{NLoS}}\left(t\right)L_{b_{m},u_{n}}^{\mathrm{NLoS}}\left(t\right)$$

Thus, the channel power gain is

(9) $$G_{b_{m},u_{n}}\left(t\right)=10^{-\frac{L_{b_{m},u_{n}}\left(t\right)}{10}}$$

Each UAV-BS is allocated bandwidth *B*. We assume different UAV-BSs operate on orthogonal frequency resources, so inter-UAV interference is ignored. Each UAV can simultaneously serve at most $N_{a}$ users. Using per-UAV FDMA, the per-user bandwidth is $B_{a}=\frac{B}{N_{a}}$. Let $P_{uav}$ denote the UAV downlink transmit power and $N_{0}$ the noise spectral density. The SNR is

(10) $$\gamma_{b_{m},u_{n}}\left(t\right)=\frac{P_{uav}G_{b_{m},u_{n}}\left(t\right)}{B_{a}N_{0}}$$

Accordingly, the achievable downlink rate is

(11) $$R_{m,n}\left(t\right)=B_{a}\log_{2}\left(1+\gamma_{b_{m},u_{n}}\left(t\right)\right)$$

The above channel model characterizes the average geometry-dependent air-to-sea link quality and is used to compute the SNR $\gamma_{b_{m},u_{n}}\left(t\right)$ and achievable rate $R_{m,n}\left(t\right)$ of each UAV–ship pair $\left(b_{m},u_{n}\right)$ at each slot *t*. The SNR threshold $\gamma_{T}$ serves as a slot-level link-feasibility criterion: UAV–ship pairs that cannot satisfy this requirement are removed from the association candidate set before PA-BAC is executed. From this perspective, communication disturbances or dynamically changing radio conditions that reduce the effective link quality can be reflected through updated SNR estimates or a more conservative choice of $\gamma_{T}$. In addition, localization errors in reported ship positions can be handled at the implementation level by using the latest estimated slot-level state, as discussed in Section 4.3. Therefore, PA-BAC only requires the current estimated link feasibility and rate information, while the strictness of the communication-feasibility condition can be adjusted through $\gamma_{T}$.

### 3.4. Mobility Model

Maritime users exhibit persistent and heterogeneous mobility. We model vessel mobility according to the priority classes defined in Section 3.2. Distress ships may lose propulsion and drift under currents and wind. We use a random walk model:

(12) $$x_{u_{n}}\left(t+1\right)=x_{u_{n}}\left(t\right)+v_{\mathrm{drift},u_{n}}\left(t\right)\cos\theta_{u_{n}}\left(t\right)$$

(13) $$y_{u_{n}}\left(t+1\right)=y_{u_{n}}\left(t\right)+v_{\mathrm{drift},u_{n}}\left(t\right)\sin\theta_{u_{n}}\left(t\right)$$

where drift direction $\theta_{u_{n}}\left(t\right)∼U\left[0,2\pi \right)$, and drift speed $v_{\mathrm{drift},u_{n}}\left(t\right)∼U\left[v_{\min}^{\mathrm{drift}},v_{\max}^{\mathrm{drift}}\right]$. Command and task users are mission-driven and typically move toward targets (e.g., distress users or designated rescue areas). Let the target position (2D) for user $u_{n}$ be $p_{\mathrm{tar},u_{n}}^{T}\left(t\right)=\left(x_{\mathrm{tar},u_{n}}^{T}\left(t\right),y_{\mathrm{tar},u_{n}}^{T}\left(t\right)\right)$. Denote the 2D position of user $u_{n}$ by $p_{u_{n}}^{T}\left(t\right)$. The update is

(14) $$p_{u_{n}}^{T}\left(t+1\right)=p_{u_{n}}^{T}\left(t\right)+v_{u_{n}}^{T}\left(t\right)\cdot \frac{p_{\mathrm{tar},u_{n}}^{T}\left(t\right)-p_{u_{n}}^{T}\left(t\right)}{∥p_{\mathrm{tar},u_{n}}^{T}\left(t\right)-p_{u_{n}}^{T}\left(t\right)∥}$$

where $v_{u_{n}}^{T}\left(t\right)\in \left[v_{\min}^{\mathrm{task}},v_{\max}^{\mathrm{task}}\right]$ is the navigation speed. When the user reaches its target within threshold ε, $∥p_{\mathrm{tar},u_{n}}^{T}\left(t\right)-p_{u_{n}}^{T}\left(t\right)∥\leq \varepsilon$, we assume it can switch to another operational target or remain in a local maneuvering mode. General users are modeled by a random waypoint process over *Z*. In each movement phase, a general user selects a random target point $p_{\mathrm{tar},u_{n}}^{R}\left(t\right)\in Z$ and a random speed $v_{u_{n}}^{R}\left(t\right)∼U\left[v_{\min}^{R},v_{\max}^{R}\right]$, then moves toward the target:


(15) $$p_{u_{n}}^{R}\left(t+1\right)=p_{u_{n}}^{R}\left(t\right)+v_{u_{n}}^{R}\left(t\right)\cdot \frac{p_{\mathrm{tar},u_{n}}^{R}\left(t\right)-p_{u_{n}}^{R}\left(t\right)}{∥p_{\mathrm{tar},u_{n}}^{R}\left(t\right)-p_{u_{n}}^{R}\left(t\right)∥}$$

If the target is reached, $∥p_{\mathrm{tar},u_{n}}^{R}\left(t\right)-p_{u_{n}}^{R}\left(t\right)∥\leq \varepsilon$, a new target and speed are selected and the process continues.

### 3.5. Energy Consumption Model

UAV-BSs are energy-constrained in maritime operations, where endurance is essential. We model UAV propulsion energy to quantify how mobility decisions translate into battery depletion. We use this energy model to construct an energy penalty term in the reward of the dynamic deployment algorithm, so that the learned policy naturally balances system utility and endurance. In each slot, the speed magnitude of UAV $b_{m}$ is

(16) $$v_{b_{m}}\left(t\right)=\frac{∥p_{b_{m}}\left(t+1\right)-p_{b_{m}}\left(t\right)∥}{\Delta t}$$

We define flight and hover indicators:

(17) $$I_{m}^{F}\left(t\right)=\left\{\begin{array}{cc} 1, & ∥p_{b_{m}}\left(t+1\right)-p_{b_{m}}\left(t\right)∥>0,\\ 0, & \mathrm{otherwise}. \end{array}\right.$$

(18) $$I_{m}^{H}\left(t\right)=\left\{\begin{array}{cc} 1, & ∥p_{b_{m}}\left(t+1\right)-p_{b_{m}}\left(t\right)∥=0,\\ 0, & \mathrm{otherwise}. \end{array}\right.$$

They satisfy the relationship

(19) $$I_{m}^{F}\left(t\right)+I_{m}^{H}\left(t\right)=1,\;\forall m\in \left[1,M\right],t\in \left[1,T\right]$$

The hovering power is

(20) $$P_{hover}=\sqrt{\frac{{\left(m_{uav}\cdot g\right)}^{3}}{2\cdot \rho_{env}\cdot S}}$$

where $m_{uav}$ is UAV mass, *g* is gravitational acceleration, $\rho_{env}$ is air density, and *S* is rotor swept area. The flying power is modeled as

(21) $$P_{fly}\left(t\right)=P_{hover}+\beta \cdot {\left(v_{b_{m}}\left(t\right)\right)}^{3}$$

where β>0 captures additional drag-related power cost. Thus, the per-slot propulsion energy consumption is

(22) $$E_{m}\left(t\right)=I_{m}^{F}\left(t\right)P_{fly}\left(t\right)\Delta t+I_{m}^{H}\left(t\right)P_{hover}\Delta t$$

The total energy consumption across all UAVs in slot *t* is

(23) $$E_{total}\left(t\right)=\sum_{m=1}^{M}E_{m}\left(t\right)$$

To reflect the physical boundary of finite battery capacity, we define cumulative energy and remaining energy:

(24) $$E_{m}^{cum}\left(t\right)=\sum_{\tau =1}^{t}E_{m}\left(\tau \right)$$

(25) $$E_{m}^{rem}\left(t\right)=E_{m}^{max}-\sum_{\tau =1}^{t-1}E_{m}\left(\tau \right)$$

where $E_{m}^{max}$ is the battery capacity of UAV $b_{m}$. In our framework, the influence of this physical limit is captured in the deployment-learning reward by penalizing propulsion energy expenditure, which encourages long-horizon endurance-aware repositioning. UAV-BSs must balance system utility with propulsion energy costs in dynamic emergencies.

### 3.6. Problem Formulation

In order to effectively deploy the emergency communication network, our goal is to jointly determine UAV deployment positions over time and per-slot user association, so as to maximize long-term system utility in a priority-sensitive maritime emergency. As emphasized in our motivation, the joint design is challenging because it couples continuous mobility decisions with discrete association indicators under strict per-slot feasibility requirements. We define the priority-weighted throughput in slot *t* as:


(26) $$H_{w}\left(t\right)=\sum_{m=1}^{M}\sum_{n=1}^{N}w\left(u_{n}\right)\cdot \alpha_{m,n}\left(t\right)\cdot R_{m,n}\left(t\right)$$

This metric rewards serving high-priority users more strongly, reflecting the urgency heterogeneity in maritime emergencies. We define the coverage ratio as the fraction of users that are associated with slot *t*:


(27) $$C\left(t\right)=\frac{\sum_{m=1}^{M}\sum_{n=1}^{N}\alpha_{m,n}\left(t\right)}{N}\in \left[0,1\right]$$

Coverage is crucial for situational awareness and broad connectivity restoration over sea areas. In maritime emergency operations, coverage reflects the ability to quickly restore basic connectivity, while priority-weighted throughput quantifies service differentiation to ensure that distress and command vessels receive preferential communication resources. To explicitly establish the optimization goal, we define the per-slot system utility strictly as the weighted sum of the coverage ratio and the normalized priority-weighted throughput. Consequently, the long-term system utility maximization problem aims to maximize this cumulative system utility over the mission horizon, formulated as

(28) $$\max\sum_{t=1}^{T}\left(\lambda_{1}C\left(t\right)+\lambda_{2}\frac{H_{w}\left(t\right)}{H_{N}}\right)$$

$$\begin{array}{cc} s.t.\;C1 & :\alpha_{m,n}\left(t\right)\in \left\{0,1\right\},\;\forall m\in M,n\in N,t\in T\\ C2 & :\sum_{n=1}^{N}\alpha_{m,n}\left(t\right)\leq N_{a},\;\forall m\in M,t\in T\\ C3 & :\sum_{m=1}^{M}\alpha_{m,n}\left(t\right)\leq 1,\;\forall n\in N,t\in T\\ C4 & :\sum_{m=1}^{M}\alpha_{m,n}\left(t\right)=1,\;\forall n\in N_{P_{1}},t\in T\\ C5 & :\alpha_{m,n}\left(t\right)\cdot \left(\gamma_{b_{m},u_{n}}\left(t\right)-\gamma_{T}\right)\geq 0,\;\forall m\in M,t\in T\\ C6 & :p_{b_{m}}\left(t\right)\in Z,\;\forall m\in M,t\in T\\ C7 & :\;∥p_{b_{m}}\left(t\right)-p_{b_{m}}\left(t-1\right)∥\;\leq \;v_{b_{m}}^{\max},\;\forall m\in M,t\in T \end{array}$$

$H_{N}$ is a fixed reference normalization factor for the priority-weighted throughput, defined as $H_{N}=NR_{\mathrm{ref}}\kappa_{H}$. Here, $R_{\mathrm{ref}}=B_{a}\log_{2}\left(1+\gamma_{T}\right)$ is the reference per-user achievable rate at the SNR threshold, and $\kappa_{H}>0$ is a dimensionless scaling coefficient. The coefficients $\lambda_{1}$ and $\lambda_{2}$ balance the coverage ratio and the normalized priority-weighted throughput, respectively.

Although the optimization variables, such as the UAV deployment positions and associations, are related, the association strategies at different time steps are independent. Given UAV deployment positions at slot *t*, the association decision only depends on instantaneous channel and capacity constraints and is therefore independent across slots. This allows us to solve the association strategy optimization problem for each time step independently. In this paper, we decompose the optimization problem into two subproblems. First, we determine the optimal association strategy based on the given UAV deployment position $p_{b_{m}}^{*}\left(t\right)$ and the user’s location. Then, we find the optimal UAV deployment position under the optimal association strategy $\alpha^{*}\left(t\right)$. We decompose the optimization problem into the following two subproblems:

(29) $$\max\frac{H_{w}\left(t\right)}{H_{N}}$$

$$\begin{array}{cc} s.t.\;C1 & :\alpha_{m,n}\left(t\right)\in \left\{0,1\right\},\;\forall m\in M,n\in N,t\in T\\ C2 & :\sum_{n=1}^{N}\alpha_{m,n}\left(t\right)\leq N_{a},\;\forall m\in M,t\in T\\ C3 & :\sum_{m=1}^{M}\alpha_{m,n}\left(t\right)\leq 1,\;\forall n\in N,t\in N\\ C4 & :\sum_{m=1}^{M}\alpha_{m,n}\left(t\right)=1,\;\forall n\in N_{P_{1}},t\in T\\ C5 & :\alpha_{m,n}\left(t\right)\cdot \left(\gamma_{b_{m},u_{n}}\left(t\right)-\gamma_{T}\right)\geq 0,\;\forall m\in M,n\in N,t\in T \end{array}$$

(30) $$\max\sum_{t=1}^{T}\left(\lambda_{1}C\left(t\right)+\lambda_{2}\frac{H_{w}\left(t\right)}{H_{N}}\right)$$

$$\begin{array}{cc} s.t.\;C1 & :p_{b_{m}}\left(t\right)\in Z,\;\forall m\in M,t\in T\\ C2 & :\;∥p_{b_{m}}\left(t\right)-p_{b_{m}}\left(t-1\right)∥\;\leq \;v_{b_{m}}^{\max},\;\forall m\in M,t\in T \end{array}$$

## 4. Proposed Algorithm

This section presents the proposed E-MAPPO-PA-BAC framework for priority-aware maritime emergency communications. The framework separates the original mixed-integer long-horizon problem into two coordinated decision layers. The first layer solves the binary user association problem at each time slot using PA-BAC, while the second layer learns continuous multi-UAV deployment through E-MAPPO. In this design, PA-BAC provides the association result used to evaluate the communication utility, and E-MAPPO updates the UAV mobility policy according to the resulting long-term reward. Therefore, the discrete association decision is handled by an optimization module, whereas the learning module focuses on continuous trajectory control.

### 4.1. PA-BAC for User Association

Given UAV positions, ship positions, and priority weights at slot *t*, PA-BAC determines the binary association variables $\alpha_{m,n}\left(t\right)$ by solving the BILP in (29) under the capacity, one-to-one association, priority-service, and SNR-feasibility constraints. The objective is to maximize the normalized priority-weighted throughput for the current slot.

The PA-BAC procedure contains three priority-aware operations. First, SNR-based screening removes infeasible UAV–ship links before optimization. Second, a priority-greedy initialization constructs an initial feasible incumbent by considering higher-priority ships first. Third, priority-guided branching preferentially selects fractional variables associated with higher-priority ships. These operations use maritime emergency priorities inside the Branch-and-Cut search, while the detailed computational cost is evaluated in Section 5.3 (Algorithm 1).
**Algorithm 1** PA-BAC for optimal user association at slot *t*.  1:**Input:** Given UAV deployment positions ${\left\{p_{b_{m}}^{*}\left(t\right)\right\}}_{m=1}^{M}$; ship positions ${\left\{p_{u_{n}}\left(t\right)\right\}}_{n=1}^{N}$; priority weights ${\left\{w\left(u_{n}\right)\right\}}_{n=1}^{N}$.  2:**Output:** Optimal association $\alpha^{*}\left(t\right)$.  3:Compute link feasibility and rates for all UAV–ship pairs using the channel model; obtain $\gamma_{b_{m},u_{n}}\left(t\right)$ and $R_{m,n}\left(t\right)$.  4:Formulate the BILP (objective (29) with constraints C1–C5).  5:Perform greedy association from higher to lower priority to obtain a feasible initial association $\alpha^{0}\left(t\right)$, and compute $H_{w}^{0}\left(t\right)$.  6:Initialize the active problem queue *Q* and push the initial BILP into *Q*. Set the current best as $\alpha^{*}\left(t\right)\leftarrow \alpha^{0}\left(t\right),H_{w}^{*}\left(t\right)\leftarrow H_{w}^{0}\left(t\right)$.  7:**while** *Q* is not empty **do**  8:      Pop and remove a subproblem I from the front of *Q*.  9:      Solve the LP relaxation of I, obtaining the relaxed solution $\alpha^{\prime }\left(t\right)$ and the objective value $H_{w}^{\prime }\left(t\right)$.10:      **if** $\alpha^{\prime }\left(t\right)$ is infeasible or $H_{w}^{\prime }\left(t\right)\leq H_{w}^{*}\left(t\right)$ **then**11:            Prune I and return to Step 7.12:      **else if** $\alpha^{\prime }\left(t\right)$ is an integer (integral) solution **then**13:            Update: $\alpha^{*}\left(t\right)\leftarrow \alpha^{\prime }\left(t\right),H_{w}^{*}\left(t\right)\leftarrow H_{w}^{\prime }\left(t\right)$, and return to Step 7.14:      **else**15:            Search for cutting planes violated by $\alpha^{\prime }\left(t\right)$ and collect them into the cut set L.16:            **if** L is not empty **then**17:                  Add L into subproblem I, and return to Step 9.18:            **else**19:                  Decompose the original problem into restricted feasible subproblems via branching; perform priority-guided branching by preferentially selecting variables corresponding to higher-priority users as branching variables; push the newly generated subproblems into queue *Q*, and return to Step 7.20:            **end if**21:      **end if**22:**end while**23:**return** $\alpha^{*}\left(t\right)$

### 4.2. E-MAPPO-PA-BAC for Dynamic Multi-UAV Deployment

The dynamic deployment subproblem is modeled as a continuous-control multi-agent decision process, where each UAV determines its movement according to the global maritime emergency state. Since the binary association variables are computed by PA-BAC, the E-MAPPO policy only needs to learn UAV mobility actions. This separation reduces the action-space complexity compared with end-to-end hybrid-action learning. The key distinction from standard MAPPO lies in the optimization-in-the-loop decision structure: at each rollout step, UAV deployment actions are evaluated after PA-BAC solves a priority-aware and constraint-satisfying association problem. Thus, the learning module is specialized for long-horizon continuous UAV mobility control, while the discrete UAV–ship association is handled by an explicit optimization layer. This design exploits the slot-wise separability of association and the long-horizon coupling of UAV movement, thereby improving feasibility, interpretability, and learning stability in maritime emergency scenarios.

Within this continuous-control layer, E-MAPPO incorporates three learning-stabilization mechanisms. First, a centralized critic estimates the value function from the global state to support cooperative credit assignment among UAVs. Second, entropy regularization is added to the actor objective to maintain exploration during training. Third, sequential actor updates with importance-weight propagation update UAV policies one by one, reducing interference among simultaneous multi-agent policy updates. These mechanisms adapt the MAPPO training backbone to the PA-BAC-in-the-loop setting, where the reward depends on global association outcomes and may change discontinuously due to SNR feasibility, capacity limits, and priority-service constraints. Together, PA-BAC-in-the-loop reward evaluation and the above stabilization mechanisms form the proposed hybrid optimization-and-learning framework.

We model the multi-UAV trajectory optimization as a discrete-time Markov decision process (MDP): $\hat{M}=\langle S,A,P,R\rangle$. To enable better deployment decisions, the policy should capture the global situation (all UAVs and ships) rather than purely local observations. Therefore, we adopt a weakly decentralized execution setting: at slot *t*, each UAV agent observes the global state as its observation: $o_{m}\left(t\right)=s\left(t\right),\forall m\in M$.

A. State space:

The global state collects UAV positions, ship positions, remaining UAV energy, and ship priority weights:


$$s\left(t\right)=[p_{b_{1}}\left(t\right),\ldots ,p_{b_{M}}\left(t\right),p_{u_{1}}\left(t\right),\ldots ,p_{u_{N}}\left(t\right),E_{1}^{rem}\left(t\right),\ldots ,E_{M}^{rem}\left(t\right),w\left(u_{1}\right),\ldots ,w\left(u_{N}\right)]$$

Here, $p_{b_{m}}\left(t\right)$ denotes the position of UAV $b_{m}$ at slot *t*, $p_{u_{n}}\left(t\right)$ denotes the position of ship $u_{n}$, $E_{m}^{rem}\left(t\right)$ is the remaining energy of UAV *m*, and $w(u_{n})$ is the priority weight of ship $u_{n}$.

B. Action space:

Each UAV is treated as an agent. Its action is defined as the UAV’s velocity along two orthogonal directions. The joint action is $a\left(t\right)=[v_{x,b_{1}}\left(t\right),v_{y,b_{1}}\left(t\right),\ldots ,v_{x,b_{M}}\left(t\right),v_{y,b_{M}}\left(t\right)]$. Moreover, the UAV’s movement at each time slot is constrained within $\left[0,v_{b_{m}}^{max}\right]$, where $v_{b_{m}}^{max}$ denotes the maximum flight speed of UAV $b_{m}$.

C. Reward design:

The per-slot reward contains three components. The first component corresponds exactly to the per-slot system utility defined in Section 3.6, combining the coverage ratio and the normalized priority-weighted throughput:


(31) $$r_{1}\left(t\right)=\lambda_{1}C\left(t\right)+\lambda_{2}\frac{H_{w}\left(t\right)}{H_{N}}$$

The second component penalizes boundary violations: if a UAV flies outside the target area *Z*, the environment returns a penalty. Let $q_{m}\left(t\right)\in \left\{0,1\right\}$ indicate whether UAV $b_{m}$ is outside *Z*; then

(32) $$r_{2}\left(t\right)=K_{1}\sum_{m=1}^{M}q_{m}\left(t\right),K_{1}<0$$

To guide the reinforcement learning process toward endurance-aware deployments, the third component penalizes energy consumption to avoid premature battery depletion:


(33) $$r_{3}\left(t\right)=-\lambda_{3}\frac{E_{total}\left(t\right)}{E_{N}}$$

Here, $E_{N}$ is the reference per-slot propulsion energy used to normalize the energy penalty. Specifically, we set $E_{N}=\sum_{m=1}^{M}\left(P_{\mathrm{hover}}+\beta {\left(v_{b_{m}}^{\max}\right)}^{3}\right)\Delta t$, which corresponds to the reference energy consumption when all UAVs fly at their maximum speeds in one slot. Therefore, in slot *t*, the total reward obtained by an agent from the environment is

(34) $$r\left(t\right)=r_{1}\left(t\right)+r_{2}\left(t\right)+r_{3}\left(t\right)$$

In this reward structure, $r_{1}\left(t\right)$ measures the true mission-level communication benefit (system utility), while $r_{2}\left(t\right)$ and $r_{3}\left(t\right)$ act as learning penalties to enforce operational safety and energy efficiency.

After collecting on-policy trajectories, E-MAPPO updates actor and critic networks. We first compute TD residuals and generalized advantage estimates (GAEs). The TD residual measures one-step surprise in value prediction:


(35) $$\delta_{j,t}=r_{j}\left(t\right)+\gamma \left(1-{done}_{j}\left(t\right)\right)V_{\phi }\left(s_{j}\left(t+1\right)\right)-V_{\phi }\left(s_{j}\left(t\right)\right)$$

A positive $\delta_{j,t}$ indicates outcomes were better than the critic predicted, while a negative value indicates worse-than-expected returns. GAEs accumulate these residuals with exponential decay to produce a low-variance, low-bias advantage estimates:


(36) $${\hat{A}}_{j,t}=\sum_{l=0}^{T-t}{\left(\gamma \lambda \right)}^{l}\delta_{j,t+l}$$

The value target used for critic regression is then

(37) $${\hat{R}}_{j,t}={\hat{A}}_{j,t}+V_{\phi }\left(s_{j}\left(t\right)\right)$$

This construction helps stabilize training by smoothing returns, especially when rewards are affected by discrete association outcomes. For actor updates, PPO relies on the likelihood ratio between the updated policy and the behavior policy used during data collection:


(38) $$r_{j,t}^{i_{\tau }}=\frac{\pi_{\theta_{i_{\tau }}}\left(a_{i_{\tau },j}\left(t\right)|s_{j}\left(t\right)\right)}{\pi_{\theta_{i_{\tau }}^{k}}\left(a_{i_{\tau },j}\left(t\right)|s_{j}\left(t\right)\right)}$$

The clipped PPO objective prevents overly large policy updates by limiting the ratio to [1−ϵ,1+ϵ]. In E-MAPPO, we additionally incorporate entropy regularization to encourage exploration:


(39) $$L\left(\theta_{i_{\tau }}\right)=\frac{1}{B_{traj}T}\sum_{j\in B}\sum_{t=1}^{T}\left[-\min\left(r_{j,t}^{i_{\tau }}M_{j,t}^{i_{\tau }},\mathrm{clip}\left(r_{j,t}^{i_{\tau }},1-\epsilon ,1+\epsilon \right)M_{j,t}^{i_{\tau }}\right)-\mu H\left(\pi_{\theta_{i_{\tau }}}\right)\right]$$

Here, $M_{j,t}^{i_{\tau }}$ denotes the sequential weights that represent the effective advantage signal when updating agent $i_{\tau }$ in sequential order, and μ controls the exploration strength. A distinctive detail is the importance-weight propagation used to correct for sequential actor updates. Since earlier agents’ policies change before later agents are updated, we propagate weights as

(40) $$M_{j,t}^{i_{\tau +1}}=\frac{\pi_{\theta_{i_{\tau }}^{new}}\left(a_{i_{\tau },j}\left(t\right)|s_{j}\left(t\right)\right)}{\pi_{\theta_{i_{\tau }}^{k}}\left(a_{i_{\tau },j}\left(t\right)|s_{j}\left(t\right)\right)}M_{j,t}^{i_{\tau }}$$

This correction reduces bias in the multi-agent gradient signal under sequential updates and mitigates destructive interference among simultaneous policy changes. Finally, the global critic is trained by minimizing the mean-squared error between predicted value and target:


(41) $$L_{V}\left(\phi \right)=\frac{1}{B_{traj}T}\sum_{j\in B}\sum_{t=1}^{T}{\left(V_{\phi }\left(s_{j}\left(t\right)\right)-{\hat{R}}_{j,t}\right)}^{2}$$

**Figure 2:**
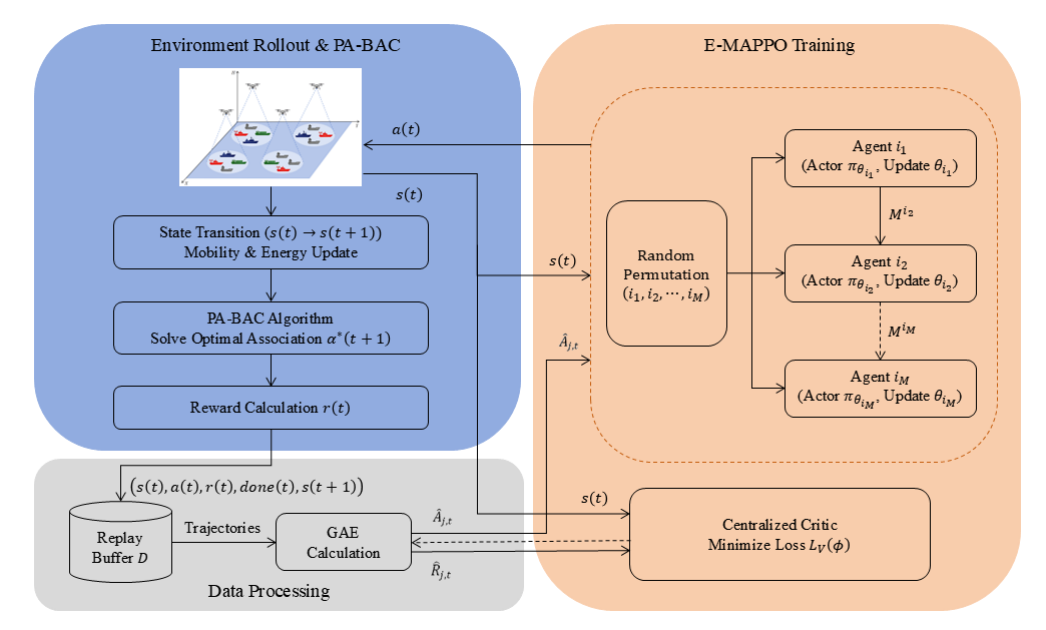
Framework of the E-MAPPO-PA-BAC algorithm.

Using a centralized critic is particularly valuable here because the reward depends on global association outcomes obtained via PA-BAC, and thus depends on the joint configuration of all UAVs and ships. The framework of the E-MAPPO-PA-BAC algorithm is illustrated in Figure 2 and Algorithm 2.
**Algorithm 2** E-MAPPO-PA-BAC training procedure.  1:**Output:** Optimal policy parameters $\Theta^{*}=\left\{\theta_{1}^{*},\ldots ,\theta_{M}^{*}\right\}$.  2:**Hyperparameters:** training iterations *K*, rollouts per iteration $B_{roll}$, horizon *T*, PPO epochs *E*, trajectories per minibatch $B_{traj}$, discount γ, GAE parameter λ, entropy coefficient μ, PPO clip ϵ.  3:Initialize *M* actor policies ${\left\{\pi_{\theta_{m}}\right\}}_{m=1}^{M}$ and a centralized critic $V_{\phi }$; set iteration k=0.  4:**while** k<K **do**  5:      Clear replay buffer *D*.  6:      Rollout: collect on-policy data.  7:      **for** rollout index j=1 to $B_{roll}$ **do**  8:            Initialize environment state $s_{j}\left(1\right)$.  9:            Run Algorithm 1 (PA-BAC) using $\left\{p_{b_{m},j}\left(1\right)\right\}$, $\left\{p_{u_{n},j}\left(1\right)\right\}$, $\left\{w\left(u_{n}\right)\right\}$ to obtain $\alpha^{*}\left(1\right)$.10:            **for** t=1 to *T* **do**11:                  Set global observation $o_{m,j}\left(t\right)=s_{j}\left(t\right)$ for all agents.12:                  Sample each agent action $a_{m,j}\left(t\right)∼\pi_{\theta_{m}^{k}}\left(\cdot |s_{j}\left(t\right)\right)$; form joint action $a_{j}\left(t\right)$.13:                  Execute actions and evolve the environment.14:                  UAV positions $p_{b_{m},j}\left(t+1\right)=p_{b_{m},j}\left(t\right)+a_{m,j}\left(t\right)\cdot \Delta t$.15:                  Update ship positions according to mobility models.16:                  Compute energy and update remaining energy according to energy model.17:                  Obtain next state $s_{j}\left(t+1\right)$, call Algorithm 1 (PA-BAC) to solve $\alpha^{*}\left(t+1\right)$ under C1–C5 and compute $H_{w}\left(t+1\right)$.18:                  Compute reward $r_{j}\left(t\right)$ by (31)–(34).19:                  Store transition $\left(s_{j}\left(t\right),a_{j}\left(t\right),r_{j}\left(t\right),{done}_{j}\left(t\right),s_{j}\left(t+1\right),\log\pi_{\theta^{k}}\left(a_{j}\left(t\right)|s_{j}\left(t\right)\right)\right)$ into *D*.20:            **end for**21:      **end for**22:      GAE: compute advantages and value targets23:      For each trajectory in *D*, compute $\delta_{j,t}$ by (35), ${\hat{A}}_{j,t}$ by (36), and ${\hat{R}}_{j,t}$ by (37).24:      **for** epoch = 1 to *E* **do**25:            Shuffle *D* and split into minibatches, each containing $B_{traj}$ trajectories.26:            **for** each minibatch B **do**27:                  Randomly generate an agent index permutation $\left(i_{1},i_{2},\ldots ,i_{M}\right)$.28:                  Initialize sequential weights $M_{j,t}^{i_{1}}={\hat{A}}_{j,t}$.29:                  **for** τ=1 to *M* **do**30:                        Compute PPO ratio $r_{j,t}^{i_{\tau }}$ by (38).31:                        Update actor parameters by minimizing $L(\theta_{i_{\tau }})$ in (39).32:                        **if** τ<M **then**33:                              update sequential weights by (40).34:                        **end if**35:                  **end for**36:                  Update critic parameters by minimizing $L_{V}\left(\phi \right)$ in (41).37:            **end for**38:      **end for**39:      Set k←k+140:**end while**

### 4.3. Practical Implementation of the Proposed Framework

To further clarify the practical feasibility of the proposed framework, we summarize here its implementation mechanism in maritime emergency scenarios. From an implementation perspective, the proposed framework can be realized by a centralized maritime emergency controller, such as a command vessel or a satellite-connected emergency coordination center. This controller is responsible for collecting the global network state and generating control decisions at each slot for the UAV fleet. Such a centralized architecture is consistent with the state definition adopted in this paper, where the decision process depends on the positions of all UAVs and ships, the remaining energy of UAVs, and the service-priority weights of ships.

At the beginning of each slot *t*, the controller collects the most recent system information, including UAV positions, ship positions, remaining UAV energy, and ship priority information. Based on the current geometry, the link-feasibility and achievable rate indicators can then be evaluated through the adopted channel and SNR model. Using this updated state, the pretrained E-MAPPO policy performs one-step online inference to generate UAV mobility actions, while PA-BAC solves the current-slot user association problem under the capacity, priority, and SNR constraints. The resulting mobility commands are then sent to the corresponding UAVs, and the association decisions are delivered to the serving UAVs, which notify ships through control signaling.

The required state information can be acquired collaboratively. UAVs periodically report their own positions and remaining energy, while ships provide location and service-priority information through onboard navigation and communication equipment, e.g., GNSS- or AIS-assisted reporting or emergency message exchange. To match the slot-based decision process, state updates are performed at slot boundaries, so that the controller always uses the latest reported state as the input of the current-slot computation. This avoids requiring continuous re-optimization within a slot and is consistent with the discrete-time model adopted in this work.

In practical maritime emergency deployments, the reported ship positions, UAV energy states, and channel-related measurements may be delayed, noisy, or partially missing, and the reported ship positions may contain localization errors. In this paper, we assume that the emergency controller uses the latest available slot-level state estimate as the input of the decision process. This assumption is consistent with AIS/GNSS-assisted maritime reporting and periodic UAV telemetry, but it does not imply perfect continuous sensing. When some state entries are delayed, noisy, or incomplete, the same framework can be combined with a state-estimation or prediction module, such as short-horizon trajectory extrapolation for ship positions, filtered telemetry for UAV energy states, and updated or filtered channel/SNR estimates for link-feasibility evaluation. This short-horizon extrapolation is particularly meaningful in maritime scenarios because vessel positions generally evolve continuously over adjacent decision slots according to navigation, rescue-task, or drift dynamics, rather than changing abruptly. Therefore, moderate AIS/GNSS reporting latency can be mitigated by propagating the latest reported ship position to the current slot using recent velocity information or the mobility model adopted in Section 3.4. Under such implementation, PA-BAC can still be executed using the estimated current state, while E-MAPPO uses the corresponding estimated global observation.

Importantly, the proposed framework does not require solving the full long-horizon mixed-integer optimization problem online at every second. Instead, the computationally intensive MARL training stage is conducted offline, whereas the online phase only involves lightweight policy inference for UAV movement and a finite-size per-slot association optimization through PA-BAC. Therefore, the framework is more practical than repeatedly solving the original coupled long-horizon problem in real time. If stricter computational constraints are considered in future engineering implementation, a two-timescale strategy can also be adopted, where UAV deployment is updated every several slots while user association is refreshed at every slot.

## 5. Simulation Results and Discussion

### 5.1. Simulation Setup

We evaluated the proposed priority-aware multi-UAV maritime emergency communication framework in maritime area *Z* over discrete time slots. Unless otherwise stated, the number of ships was fixed at N=50, and the number of UAVs varied as M∈{3,4,5,6} to examine scalability. UAVs provide downlink access to ships under practical air-to-sea propagation and service constraints, where each UAV can serve only a limited number of ships per slot and a link is feasible only if the received SNR exceeds a predefined threshold. Ships are categorized into four priority levels (higher weight indicates higher urgency), and heterogeneous mobility is considered to capture realistic maritime dynamics, including distress drifting, destination-driven mission ships, and random-mobility general ships. The main simulation parameters are summarized in Table 1.

We evaluate two coupled aspects: (i) the per-slot association solver PA-BAC versus two priority-based heuristics (P-Greedy and P-Nearby) under the normalized priority-weighted throughput metric; (ii) the long-horizon deployment policy E-MAPPO-PA-BAC versus MARL baselines (MAPPO and Multi-Agent Deep Deterministic Policy Gradient (MADDPG)) in terms of long-term system utility. In addition, since the learning reward incorporates energy penalties to guarantee practical endurance, we further evaluated the energy consumption per unit of system utility and tested the algorithm’s robustness across different user density scenarios.

### 5.2. Parameter Sensitivity Analysis

To further examine the sensitivity of the proposed framework to key modeling parameters, we conducted additional parameter-sensitivity experiments. These experiments evaluated how the proposed E-MAPPO-PA-BAC framework responds to changes in the utility-weight setting and communication-feasibility condition. In particular, varying the SNR threshold reflects different levels of link-quality requirements and provides a conservative way to test the framework under more or less stringent radio conditions, such as when additional link margins are needed to cope with communication disturbances or time-varying channel quality.

**Figure 3:**
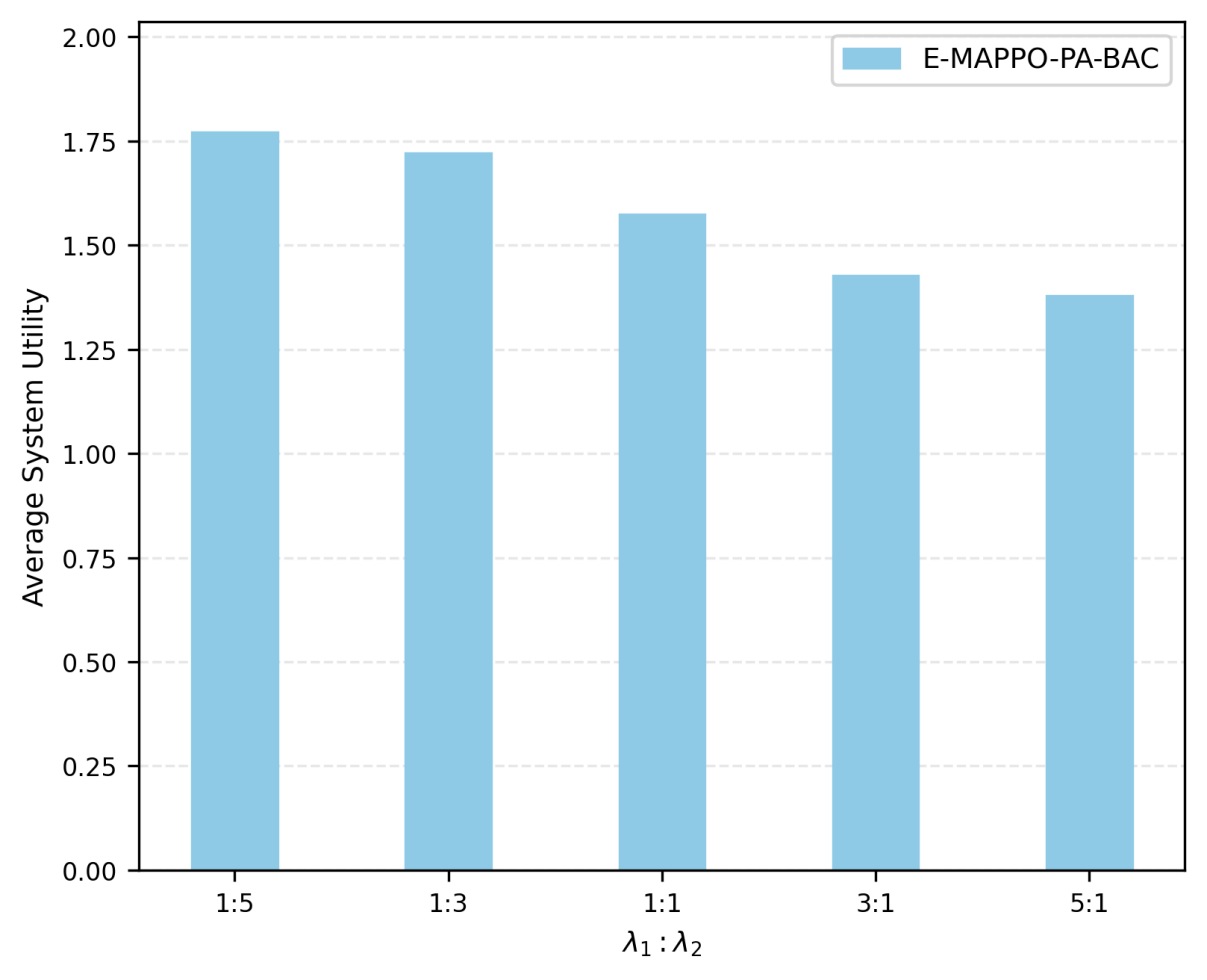
Impact of the weight-coefficient ratio $\lambda_{1}:\lambda_{2}$ on average system utility.

Figure 3 shows the impact of the weight-coefficient ratio $\lambda_{1}:\lambda_{2}$ on the average system utility of E-MAPPO-PA-BAC. The results show that the average system utility is higher when a larger weight is assigned to the normalized priority-weighted throughput term. This is because, under the default capacity-limited setting, the achievable coverage is constrained by the maximum number of users that can be served by the UAV fleet, whereas the priority-weighted throughput term more directly reflects the benefit of serving high-priority ships through high-quality links. Therefore, $\lambda_{1}$ and $\lambda_{2}$ provide a tunable mechanism for adapting the objective to different emergency-service preferences.

**Figure 4:**
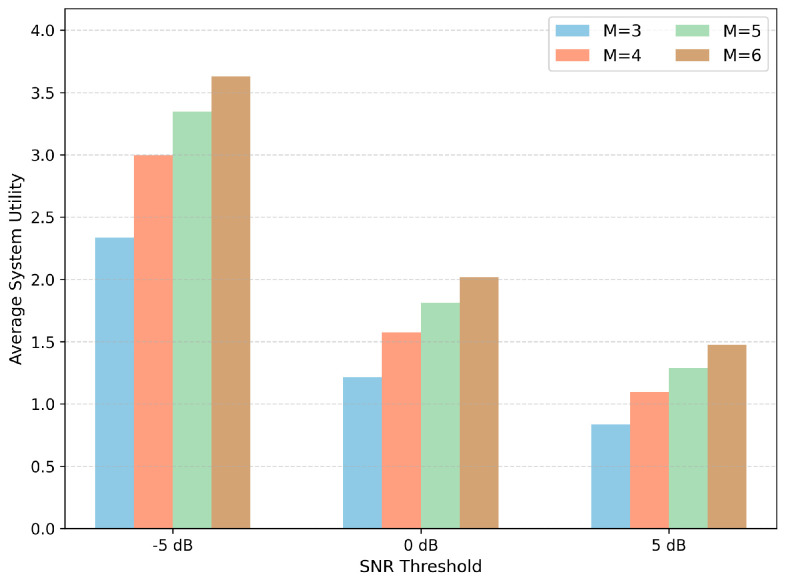
Average system utility under different SNR thresholds and numbers of UAVs.

Figure 4 evaluates the average system utility under different SNR thresholds and UAV fleet sizes. A higher SNR threshold imposes a stricter link-feasibility requirement, which reduces the number of feasible UAV–ship links and makes the association problem more constrained. As a result, the average system utility decreases as the SNR threshold increases from −5 dB to 5 dB. For all tested SNR thresholds, increasing the number of UAVs improves the system utility because more UAVs provide larger spatial coverage and more association opportunities. These results indicate that E-MAPPO-PA-BAC remains effective under different communication-feasibility requirements, while also showing the expected degradation under stricter radio-link conditions.

**Figure 5:**
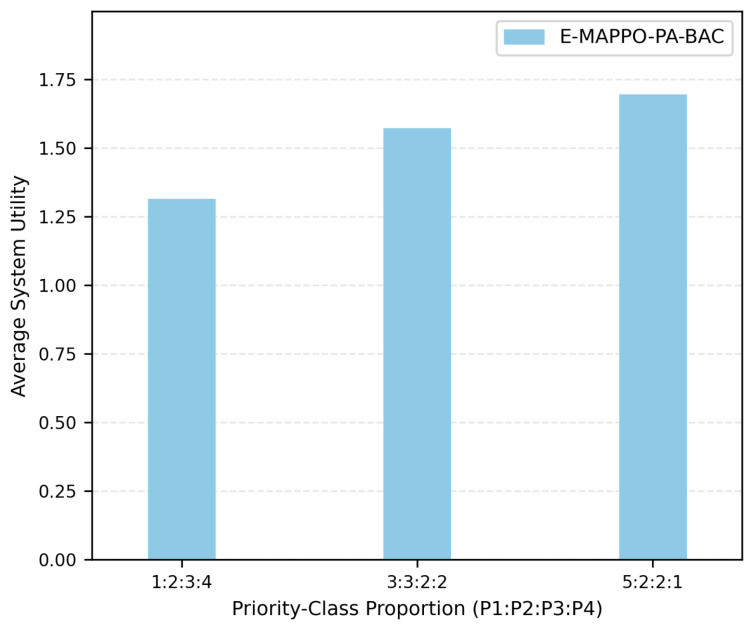
Impact of priority-class proportion on average system utility.

Figure 5 shows the impact of different priority-class proportions on the average system utility. The tested ratios represent different emergency compositions, ranging from scenarios with more low-priority users to scenarios with a larger proportion of high-priority users. The results show that the average system utility changes with the user-priority composition. When the proportion of high-priority users increases, PA-BAC allocates more resources to ships with urgent needs according to the priority-weighted objective, leading to a higher weighted utility. This result confirms that the proposed framework is sensitive to emergency-priority composition and can adapt the association decision to different rescue-task requirements.

### 5.3. Ablation Study and Computational Efficiency

To further clarify the individual contribution of each key design component, we conducted an ablation study on the proposed E-MAPPO-PA-BAC framework. Specifically, under the same evaluation setting with N=50 and M∈{3,4,5,6}, we compared the full framework with three degraded variants: w/o PA-BAC, where the PA-BAC module is removed; w/o Entropy, where the entropy regularization term is removed from the actor objective; and w/o Sequential, where the sequential actor updates are disabled. We evaluate these variants in terms of average system utility and average energy consumption per system utility.

**Figure 6:**
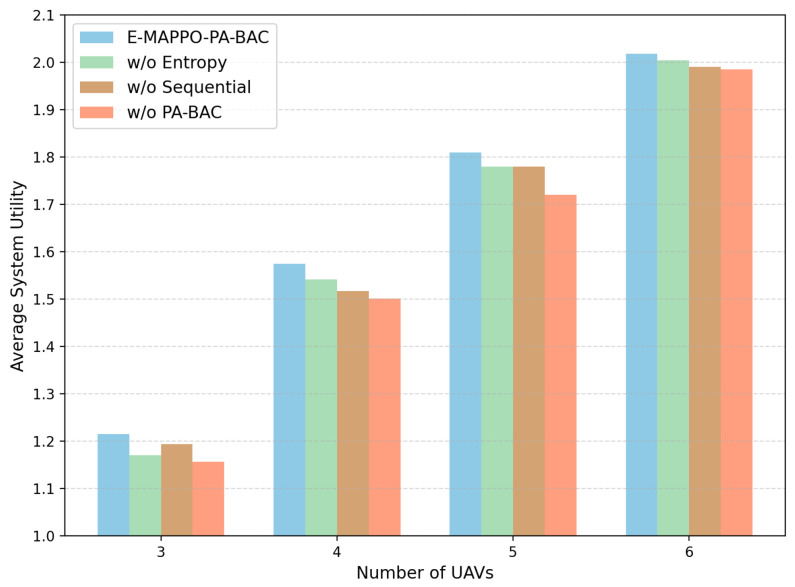
Ablation study on average system utility under different numbers of UAVs.

Figure 6 shows that the full E-MAPPO-PA-BAC framework consistently achieves the highest average system utility across all tested fleet sizes. Removing PA-BAC causes the largest performance degradation, which indicates that exact and constraint-aware slot-level association is the most critical component for improving the long-term utility. Removing entropy regularization or sequential actor updates also reduces the achieved utility, although the impact is comparatively smaller. This suggests that entropy regularization helps maintain effective exploration during training, while sequential actor updates improve coordination stability among UAV agents in the multi-agent learning process.

**Figure 7:**
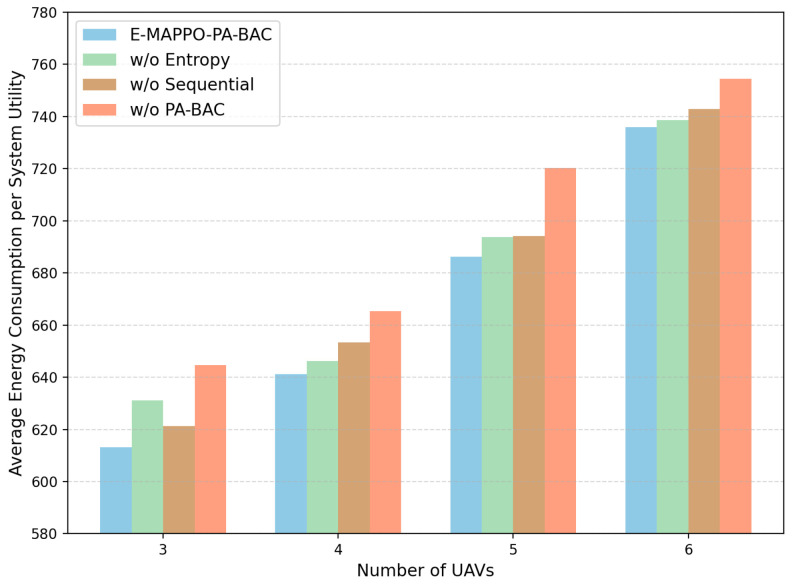
Ablation study on average energy consumption per system utility under different numbers of UAVs.

Figure 7 further reports the average energy consumption per system utility for the same ablation variants. The full E-MAPPO-PA-BAC framework consistently yields the lowest energy consumption per unit utility in all tested settings, showing that the proposed design not only improves long-term communication performance but also maintains better energy efficiency. In particular, removing PA-BAC results in the highest energy cost per unit utility, which indicates that the absence of accurate slot-level association may induce less efficient long-horizon UAV deployment behaviors. Taken together, Figure 6 and Figure 7 confirm that PA-BAC, entropy regularization, and sequential actor updates all contribute positively to the final performance, with PA-BAC playing the dominant role.

Since the PA-BAC algorithm is executed at every time slot, its computational cost deserves further clarification. Let $F_{t}$ denote the number of feasible UAV–ship links after SNR-based feasibility screening at slot *t*, where $F_{t}\leq MN$. Computing link feasibility and achievable rates for all candidate links requires O(MN) operations. Constructing the priority-greedy initial incumbent requires approximately O(MN+NlogN) operations, while building the BILP model requires $O(F_{t}+M+N)$. The subsequent Branch-and-Cut search has exponential worst-case complexity with respect to the number of feasible binary variables, i.e., $O(2^{F_{t}})$.

In practice, however, the effective runtime is much lower than this worst-case bound. First, SNR-based feasibility screening removes a large number of infeasible UAV–ship pairs before optimization. Second, the priority-greedy initialization quickly provides a feasible incumbent, which improves the lower bound and accelerates pruning. Third, priority-guided branching tends to explore more promising subproblems earlier, further reducing the effective search space in practical solving.

**Figure 8:**
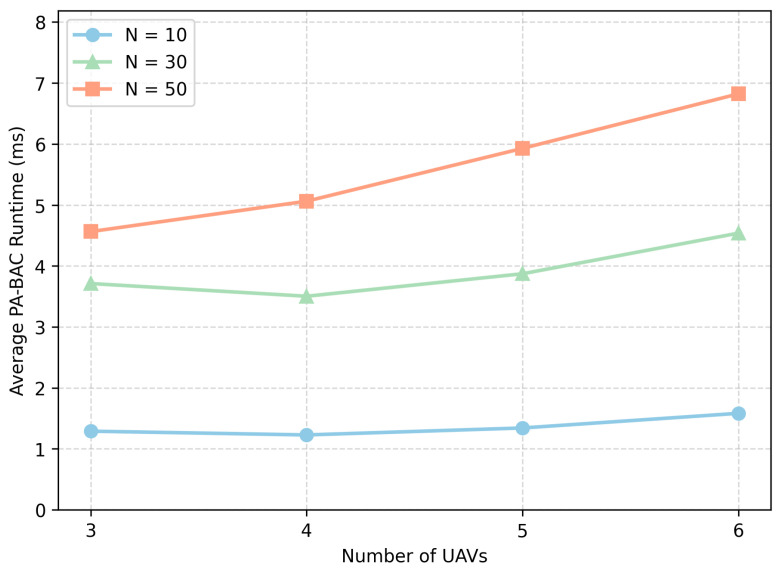
Average runtime of PA-BAC for single-slot association decisions under different numbers of UAVs and users.

To further validate the practical efficiency of PA-BAC, we measured its pure wall-clock runtime for single-slot association decisions under different problem sizes. Specifically, we tested M∈{3,4,5,6} and N∈{10,30,50} over repeated random instances, and recorded the average runtime in milliseconds. The profiling was conducted on an otherwise idle local machine to reduce interference from unrelated background processes and better reflect the execution time of the PA-BAC module itself. As shown in Figure 8, the average runtime generally increases with the problem size, but remains at a very low level in all tested configurations. Even in the largest tested case, the average runtime is below approximately 7 ms. Since the slot duration in our system is Δt=1 s, the runtime of PA-BAC occupies less than 0.7% of one decision interval. This confirms that embedding PA-BAC into each time slot is computationally practical for the considered maritime emergency deployment setting.

### 5.4. Effectiveness of Priority-Aware Association and Hybrid Deployment

**Figure 9:**
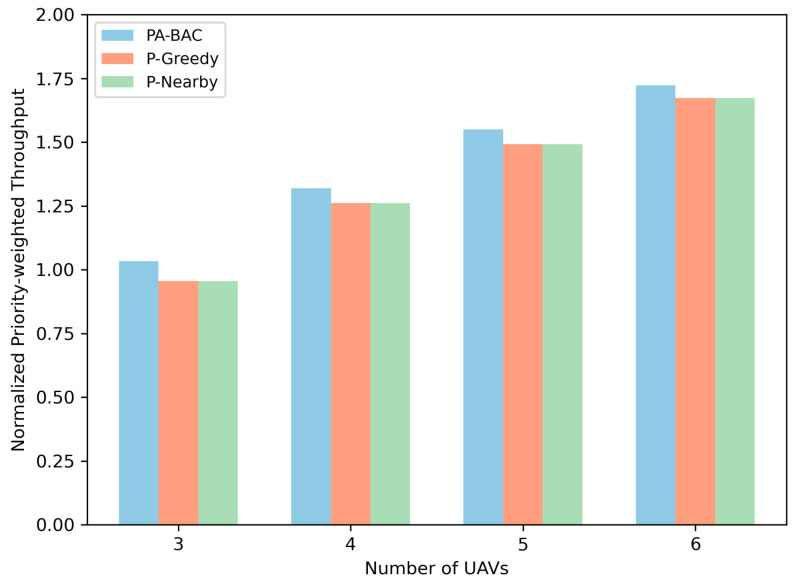
Comparison of different association policies.

Figure 9 compares the normalized priority-weighted throughput $H_{w}\left(t\right)/H_{N}$ achieved by PA-BAC against P-Greedy and P-Nearby when N=50 is fixed and *M* varies from 3 to 6. The key outcome is consistent across all fleet sizes: PA-BAC yields the highest normalized priority-weighted throughput, and the advantage persists as the number of UAVs increases. A heuristic can satisfy priorities in a superficial sense (e.g., always trying to assign $P_{1}$ first), yet still be inefficient in how it uses the remaining capacity and in how it resolves conflicts created by capacity limits $N_{a}$ and the one-to-one association rule. PA-BAC is designed exactly for this coupled structure: it solves the BILP using LP relaxation bounds, cutting planes, and branching (guided by emergency priorities), enabling it to revisit and correct early greedy decisions that later prove globally suboptimal. The two heuristics fail for different structural reasons. P-Nearby emphasizes short distance, which typically improves SNR and thus instantaneous rate. However, in a multi-UAV multi-priority setting, choosing “nearest” can concentrate many ships onto the same UAV (especially when ships are clustered), causing capacity saturation and leaving other UAVs underutilized. This harms the weighted objective because it may allocate scarce capacity to low-priority ships that are nearby while forcing high-priority ships to associate with suboptimal UAVs. P-Greedy, on the other hand, explicitly prioritizes high-weight ships but remains myopic: once it assigns a ship to a UAV, it typically does not reconsider that assignment even if later ships (still high-priority) would have benefited more from that UAV due to SNR feasibility or marginal throughput gains. This “capacity blocking” effect becomes more pronounced when $N_{a}$ is tight. This indicates that, under strict emergency priority stratification, PA-BAC can rigorously protect high-urgency rescue operations while utilizing limited UAV capacity efficiently.

**Figure 10:**
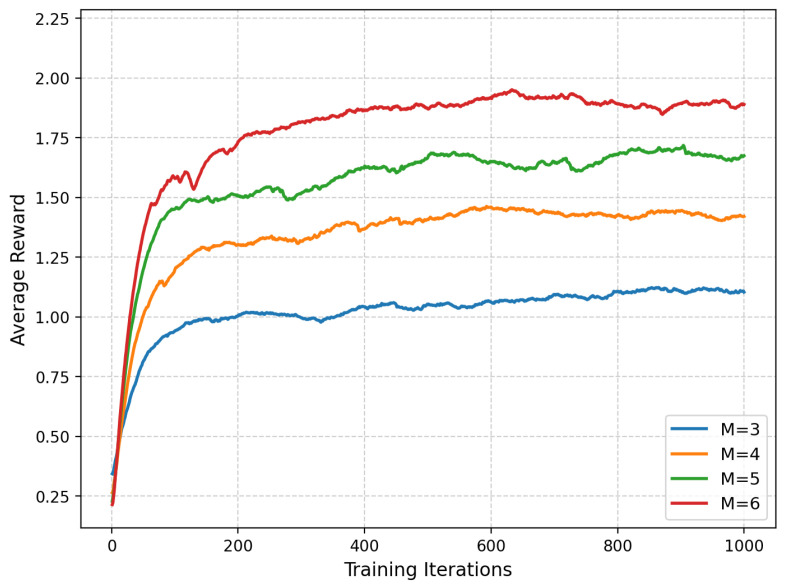
Learning curves of E-MAPPO-PA-BAC under different numbers of UAVs.

Figure 10 plots the learning curves (average reward) of E-MAPPO-PA-BAC for M∈{3,4,5,6} UAVs under the same training budget and network size. First, the training curves exhibit fast early improvement followed by stable convergence with limited oscillation. This behavior is nontrivial in cooperative MARL because each agent’s policy update changes the environment perceived by others (non-stationarity), which often causes reward swings. Here, stability is largely attributed to the hybrid design: the discrete association is not “implicitly learned” but computed each step by PA-BAC, which makes the reward less noisy and more aligned with the true system objective. In other words, the deployment policy receives feedback evaluated under a consistent association mechanism instead of a fluctuating heuristic. This improves the signal-to-noise ratio of policy gradients and reduces the frequency of abrupt reward drops that would otherwise be triggered by temporary priority violations or infeasible assignments during exploration. Second, the asymptotic reward increases monotonically with *M*. This is expected because adding UAVs increases the system’s instantaneous service capacity and improves spatial degrees of freedom: more UAVs can simultaneously cover multiple ship clusters that emerge under heterogeneous mobility. Importantly, the improvement is not merely due to serving “more ships”; the learning reward integrates the per-slot system utility (coverage rate and normalized priority-weighted throughput) along with energy and boundary penalties. Thus, higher rewards indicate that the learned deployment is exploiting additional UAV resources to both keep more ships above the SNR threshold and allocate capacity to high-priority ships in a way that improves the weighted throughput term. Third, the convergence speed does not degrade sharply as *M* increases from 3 to 6. In principle, more agents enlarge the joint action space and complicate coordination. The fact that convergence remains efficient suggests that the “learning burden” is effectively reduced by decomposition: the continuous control policy focuses on positioning and mobility, while the combinatorial association decisions are handled by PA-BAC, which enforces feasibility with respect to capacity, SNR threshold, and mandatory service to distress ships.

**Figure 11:**
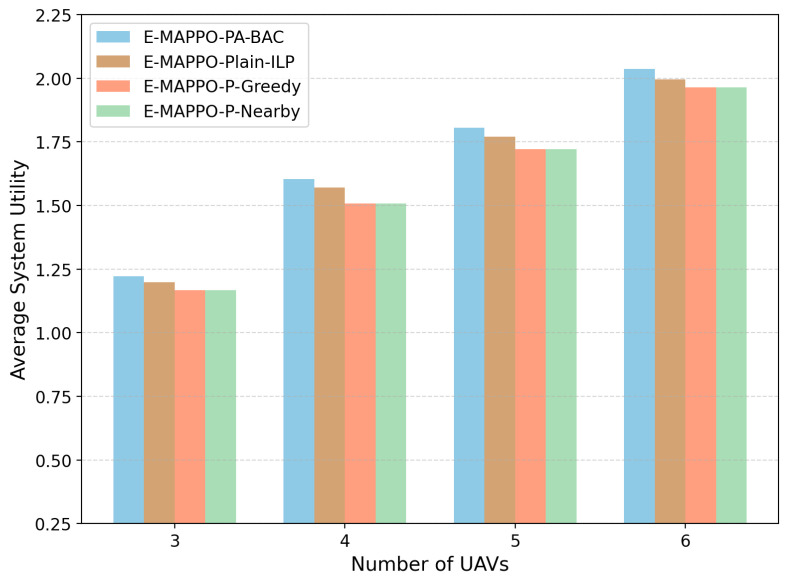
Average system utility under different association modules with the same E-MAPPO deployment policy.

Figure 11 evaluates the average long-term system utility achieved by E-MAPPO when it is paired with different association modules, including PA-BAC, Plain-ILP, P-Greedy, and P-Nearby. Here, E-MAPPO-P-Greedy and E-MAPPO-P-Nearby represent RL-plus-heuristic hybrid baselines, where the same E-MAPPO deployment policy is combined with heuristic association rules. E-MAPPO-Plain-ILP represents an RL-plus-ILP baseline, where the same association BILP is solved by a generic ILP solver without the PA-BAC-specific priority-greedy initialization and priority-guided branching enhancement. Therefore, this comparison isolates the impact of different association modules under the same learned deployment policy. The results show that E-MAPPO-PA-BAC consistently achieves the highest average system utility across all tested UAV fleet sizes. E-MAPPO-Plain-ILP achieves a utility close to E-MAPPO-PA-BAC because both methods solve the same association BILP. However, PA-BAC provides a slightly higher utility under the per-slot time-limited setting due to its priority-aware feasible initialization and priority-guided branching. Compared with E-MAPPO-P-Greedy and E-MAPPO-P-Nearby, both ILP-based methods achieve higher system utility, confirming the benefit of global association optimization under capacity, priority, and SNR constraints. These results demonstrate that the performance gain of the proposed framework does not only come from the E-MAPPO deployment policy, but also from the priority-aware optimization layer used for per-slot association.

### 5.5. Comparison with MARL Baselines and Energy Efficiency

**Figure 12:**
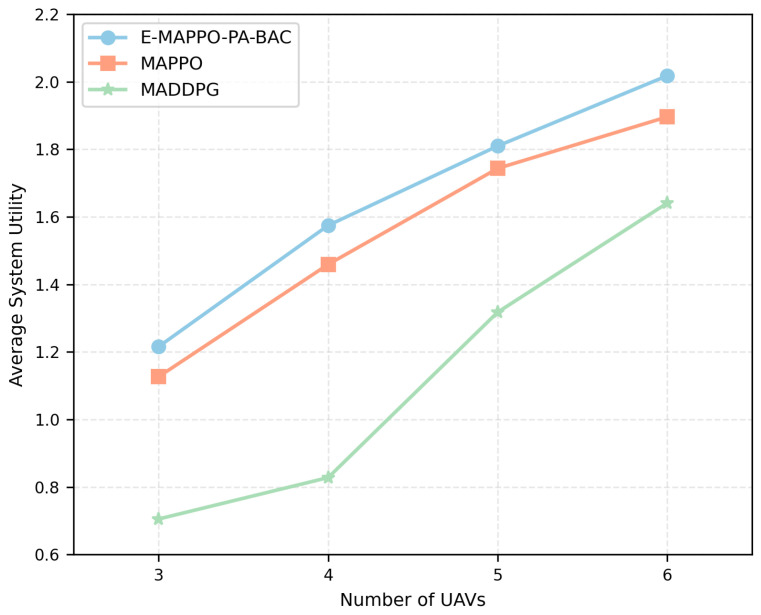
Average system utility under different multi-agent learning algorithms.

Figure 12 compares E-MAPPO-PA-BAC with MAPPO and MADDPG under the same evaluation settings (N=50,M∈{3,4,5,6}), using average system utility as the metric. The proposed framework achieves the best performance for all tested fleet sizes, and the gap generally becomes more pronounced as *M* increases. The advantage over standard MAPPO can be understood through the enhancement mechanisms introduced in E-MAPPO. In maritime emergency settings, ship mobility is heterogeneous and non-stationary, and the reward depends on both continuous deployment actions and hard-threshold events. Such environments amplify critic variance and worsen credit assignment across agents. E-MAPPO addresses this by leveraging a global value network (conditioning value estimation on the global state), entropy regularization (preventing premature collapse to overly conservative deployments), and sequential actor updates (reducing gradient interference among agents). These design choices are particularly beneficial when *M* is larger because coordination complexity increases and simultaneous updates can destabilize learning. MADDPG underperforms more significantly because deterministic policy gradients are typically more sensitive to non-stationarity and to discontinuous rewards. In this problem, coverage changes discretely when links cross the SNR threshold, and association feasibility is constrained by binary decisions and mandatory service rules. Such characteristics can induce sharp changes in observed returns, which makes critic learning harder and can lead to brittle policies that do not generalize well across different ship configurations. In contrast, PPO-style clipped objectives and advantage-based updates (as used in E-MAPPO) are empirically more robust to these instabilities. This confirms that advantage-based updates and global value networks are crucial for learning robust UAV deployments in highly dynamic maritime emergencies.

**Figure 13:**
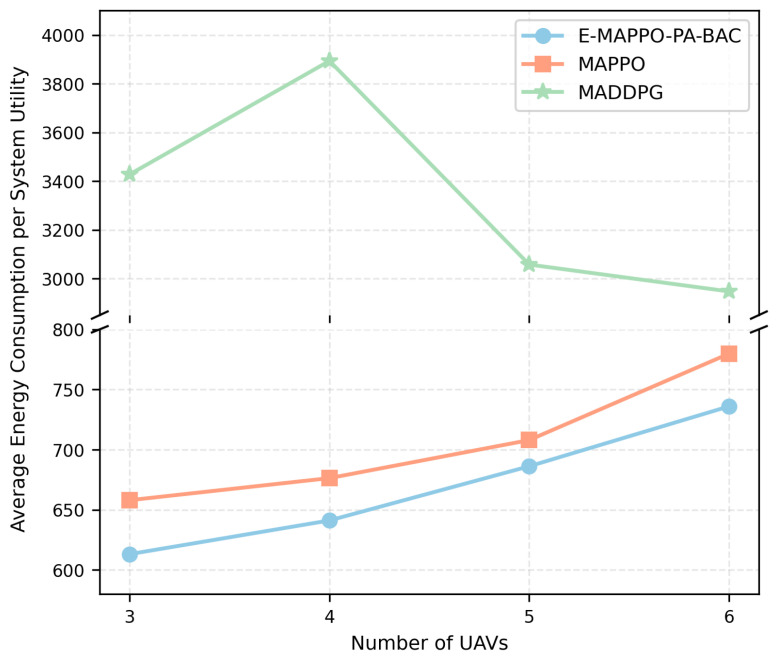
Average energy consumption per system utility under different multi-agent learning algorithms.

Figure 13 compares the energy efficiency of E-MAPPO-PA-BAC, MAPPO, and MADDPG using the metric of average energy consumption per unit system utility as the number of UAVs increases. The results indicate that E-MAPPO-PA-BAC consistently achieves the lowest energy consumption per unit utility across all fleet sizes, meaning that it delivers higher system utility with less propulsion cost. As *M* grows, the performance gap between E-MAPPO-PA-BAC and MAPPO remains stable, suggesting that the proposed sequential actor updates and the PA-BAC-in-the-loop association enable scalable coordination without inducing excessive repositioning. In contrast, MADDPG exhibits substantially higher energy consumption per utility, implying less energy-efficient movement decisions under discontinuous, constraint-sensitive rewards. This advantage is particularly important for maritime emergency communications, where UAV endurance directly determines how long reliable connectivity can be sustained during time-critical search-and-rescue missions.

**Figure 14:**
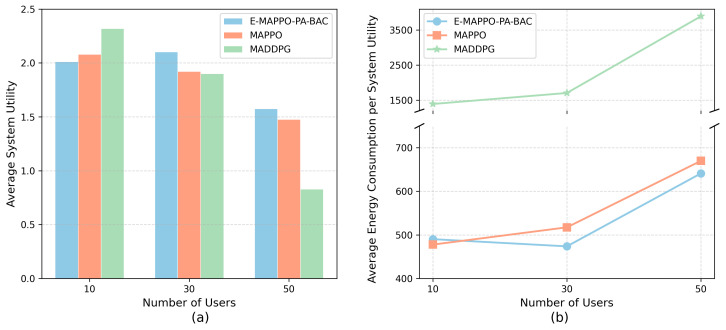
Impact of user density (number of ships) on (**a**) average system utility and (**b**) average energy consumption per system utility.

Figure 14 evaluates robustness under varying user densities by changing the number of ships (N∈{10,30,50}) with a fixed fleet of four UAVs, reflecting different stages of maritime emergency operations from a sparse initial search to a congested rescue phase. When N=10, the system is lightly loaded and UAV capacity is relatively abundant; therefore, heuristic association and baseline policies can already satisfy most demands, yielding comparable average system utility that is occasionally slightly higher, as well as marginally lower energy consumption per unit utility. This suggests that the proposed framework may trade a small performance margin in easy regimes for stricter feasibility and priority consistency. Importantly, as the user density increases (N∈{30,50}), capacity constraints and priority conflicts become pronounced. In these more critical and congested regimes, E-MAPPO-PA-BAC consistently achieves higher system utility and better energy efficiency than MAPPO and MADDPG, indicating stronger robustness under stress. By coupling MARL-based deployment with PA-BAC to generate feasible, priority-aware associations under capacity and SNR constraints, the proposed framework maintains reliable performance when the network is heavily loaded, confirming its suitability for unpredictable and time-critical maritime emergency communications.

### 5.6. AIS-Driven Mobility Trace Evaluation and Comparison

**Figure 15:**
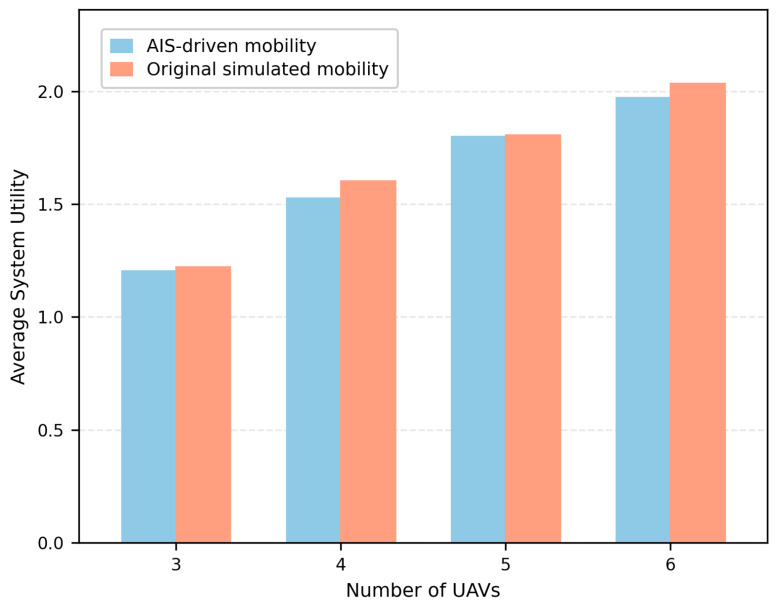
Average system utility under AIS-driven and original simulated mobility with different numbers of UAVs using the proposed E-MAPPO-PA-BAC framework.

Figure 15 further evaluates the proposed E-MAPPO-PA-BAC framework under AIS-driven realistic ship mobility traces and compares it with the original simulated mobility setting. In the AIS-driven evaluation, public AIS ship trajectories are used to replace the synthetic ship mobility model, while the priority labels, communication model, and PA-BAC association constraints remain consistent with the main simulation setting. The average system utility increases with the number of UAVs under both mobility settings, indicating that the proposed framework benefits from additional UAV spatial resources in both synthetic and realistic mobility scenarios. Compared with the original simulated mobility setting, the AIS-driven mobility results are slightly lower or comparable across the tested UAV fleet sizes. This is reasonable because real AIS traces may introduce more irregular and less controllable vessel distributions than the synthetic mobility model. Nevertheless, the AIS-driven results follow the same increasing trend and remain close to those obtained under the original simulated mobility setting, which further supports the applicability of the trained E-MAPPO-PA-BAC framework under realistic ship mobility patterns.

## 6. Conclusions

This paper studied UAV-enabled maritime emergency communications with heterogeneous service priorities. We formulated a long-horizon system-utility maximization problem that jointly optimizes per-slot user association and dynamic multi-UAV deployment under capacity, priority, SNR, mobility, geofencing, and energy constraints. To address the coupled discrete–continuous nature of the decision variables, we proposed a hybrid optimization-and-learning framework that combines a Priority-Aware Branch-and-Cut (PA-BAC) method for association and an Enhanced MAPPO (E-MAPPO) method for deployment. PA-BAC leverages LP relaxation, cutting planes, and priority-guided branching to generate high-quality feasible associations, while E-MAPPO employs a global value network, entropy regularization, and sequential actor updates to improve multi-agent learning stability. Simulation results showed that PA-BAC outperforms heuristic association baselines in normalized priority-weighted throughput. Furthermore, the integrated E-MAPPO-PA-BAC framework not only achieves higher long-term system utility and better scalability than MAPPO and MADDPG, but also exhibits superior energy efficiency and strong robustness across both sparse and dense maritime user distributions. Additional ablation, runtime, sensitivity, and AIS-driven trace evaluations further verified the contribution of each component, the computational practicality of the PA-BAC module, and the applicability of the proposed framework under realistic ship mobility patterns. Future work will extend the framework to integrated space–air–sea emergency networks by coordinating UAVs with satellite systems and unmanned surface vehicles (USVs), and will investigate the joint optimization of communication, sensing, and computing tasks for comprehensive emergency operations. Another important future direction is to develop robust and partially observable extensions of the proposed framework that can explicitly account for delayed AIS/GNSS updates, localization errors, channel-estimation uncertainty, stochastic communication disturbances, and dynamically changing radio conditions.

## Figures and Tables

**Table 1:** Main simulation parameters.

Parameter	Value
Episode length (*T*)	300
Carrier frequency ($f_{c}$)	2 GHz
Bandwidth per UAV (*B*)	10 MHz
UAV altitude (*h*)	100 m
UAV transmit power ($P_{uav}$)	1 W
Maximum number of users served per UAV ($N_{a}$)	8
SNR threshold ($\gamma_{T}$)	0 dB
Noise power spectral density ($N_{0}$)	−174 dBm/HZ
User priority weights	4.0, 3.0, 2.0, 1.0
Proportion of users in each priority level	3:3:2:2
Maximum UAV flight speed ($v_{b_{m}}^{max}$)	20 m/s
Actor learning rate	0.0003
Critical learning rate	0.0003
Discount factor (γ)	0.99
GAE parameter (λ)	0.95
Training iterations (*K*)	1000

## Data Availability

The data that support the findings of this study are available from the corresponding author upon reasonable request.
